# *PGC-1alpha *as modifier of onset age in Huntington disease

**DOI:** 10.1186/1750-1326-4-10

**Published:** 2009-02-06

**Authors:** Elahe Taherzadeh-Fard, Carsten Saft, Jürgen Andrich, Stefan Wieczorek, Larissa Arning

**Affiliations:** 1 Department of Human Genetics, Ruhr-University, 44780 Bochum, Germany; 2 Department of Neurology, St. Josef-Hospital, Ruhr-University, 44791 Bochum, Germany

## Abstract

Although there is a strong correlation between CAG repeat length and age at onset (AO) of motor symptoms, individual Huntington disease (HD) patients may differ dramatically in onset age and disease manifestations despite similar CAG repeat lengths. This has led to a search for genetic factors that influence AO. In order to identify such a genetic modifier, we analysed polymorphisms in the *PGC-1alpha *gene. Recent data indicate inhibition of PGC-1alpha function by mutant Htt supporting a link between transcriptional deregulation and mitochondrial dysfunction in HD. In > 400 HD patients, a polymorphism located within intron 2, a potential recombination hot spot, explains a small, but statistically significant, amount of the variability in AO. Our data suggest that PGC-1alpha has modifying effects on the pathogenic process in HD.

## Findings

Huntington Disease (HD) is an autosomal-dominant disorder due to lesions in the striatum that cause involuntary choreiform movements and progressive behavioral and cognitive impairment. The underlying mutation is an expansion of an unstable CAG repeat in the *HD *gene resulting in an expanded polyglutamine tract in huntingtin protein (Htt) [1]. HD shows highly variable clinical expression, as exemplified by the wide variation of AO. The strong inverse relationship between AO and number of CAG repeats is well-defined. Yet, there is substantial variation in AO that is not explained by the HD repeat [2-6].

To date, several genetic modifiers of HD have been described in independent studies. All of these modifiers relate to various mechanisms implicated in HD pathology such as excitotoxicity, dopamine toxicity, metabolic impairment, transcription deregulation, protein misfolding and oxidative stress [5,7-11]. Additionally, genomewide linkage scans revealed potential loci that may contain genes that modify AO [12-14].

Increasing evidence implicates mitochondrial dysfunction and metabolic impairment in HD pathology [for review see [15]]. In particular, PGC-1alpha (peroxisome proliferator-activated receptor [PPAR]-g coactivator 1a) as a key transcriptional co-regulator is an important mediator in protecting neurons against oxidative damage and seems to be involved in HD pathogenesis. PGC-1alpha induces the transcription of cellular programs regulating mitochondrial respiration, oxidative stress defense and adaptive thermogenesis [16]. Recent data indicate inhibition of PGC-1alpha function by mutant Htt supporting a link between transcriptional deregulation and mitochondrial dysfunction in HD [17-19]. Altered PGC-1alpha function may, therefore, contribute to HD pathogenesis.

A total of 15 single nucleotide polymorphisms (SNPs) in the peroxisome proliferators-activated receptor γ coactivator 1 α (PPARGC1A) gene (rs2970865, rs2970866, rs4383605, rs2946386, rs2970869, rs17576121, rs2970870, rs7695542, rs2970873, rs2946385, rs12374310, rs7665116, rs2970855, rs2970848, rs8192678) were selected for genotyping in a German HD cohort of more than 400 unrelated patients recruited from the Huntington Center NRW in Bochum. Clinical assessment and determination of the motor AO was performed exclusively by experienced neurologists of the Center. The expanded CAG repeats ranged from 40 to 66 trinucleotide units and AO ranged from 16 to 76 years of age, with a mean of 45 years. *HD *CAG repeat sizes were determined by polymerase chain reaction using an assay counting the perfectely repeated (CAG)_n _units. Informed consent was obtained from all patients and controls. The studies were performed in a manner that fully complies with the Code of Ethics of the World Medical Association (Declaration of Helsinki) and was approved by the relevant university review board.

The polymorphisms were selected from NCBI SNP database due to their potential functional relevance, *e.g. *polymorphisms in the 5'-UTR were included due to their potential to influence gene expression, and their relative frequency. The genotype distributions of all the chosen polymorphisms were consistent with Hardy-Weinberg equilibrium (HWE).

Controlling for the effect of CAG repeat length on AO revealed an R^2 ^value of 0.729 indicating that nearly 73% of the variation in AO could be explained by the mutation itself (Table 1). In addition to the number of the expanded CAG repeats, the modifying effects of the polymorphisms in *PGC-1alpha *on the AO were examined.

**Table 1:** Variability in AO attributable to the CAG repeat length was assessed by linear regression using the logarithmically transformed AO as the dependent variable and SNP genotypes as independent variables.

**Model**		**R^2^**	**ΔR^2^**	**% additionally explained variance**	***P *value**
HD CAG 40–66 (n = 401)		0.729	-		< 0.0005

***PGC-1alpha *Polymorphis ms**					

rs2970865	promoter region	-	-	-	-
rs2970866	promoter region	-	-	-	-
rs4383605	promoter region	-	-	-	-
rs2946386	promoter region	-	-	-	-
rs2970869	promoter region	-	-	-	-
rs17576121	promoter region	-	-	-	-
rs2970870	promoter region	-	-	-	-
rs7695542	promoter region	-	-	-	-
rs2970873	intron1	-	-	-	-
rs2946385	intron2	-	-	-	-
rs12374310	intron2	-	-	-	-
**rs7665116**	**intron2**				
**0,1,2**		**0.732**	**0.003**	**1.1**	**0.025**
**0,1,1**		**0.733**	**0.004**	**1.5**	**0.012**
rs2970855	intron5	-	-	-	-
rs2970848	intron7	0.731	-	-	0.054
rs8192678	exon 8	-	-	-	-

R^2 ^illustrates the relative improvement of the regression model when the genotypes are considered in addition to the CAG repeats; ΔR^2 ^values quantify these differences. (-) indicates no increase in R^2^.

Here, we saw evidence of association of the rs7665116 SNP. The R^2 ^statistic rose modestly but significantly (from 0.729 to 0.732, p = 0.025 in the additive model, TT vs TC vs CC, and from 0.729 to 0.733, p = 0.012, in the dominant model, TT vs TC+CC) when rs7665116 genotypes were added to the regression model (Table 1). The mean AO in patients homozygous for the wildtype allele T is 45.08 years of age, while the mean AO for patients homozygous for the C allele is 47.3 years of age. SNP rs2970848 in intron 7 shows a trend towards association, for all other polymoprhism no impact on the R^2 ^statistic could be observed.

**Figure 1:**
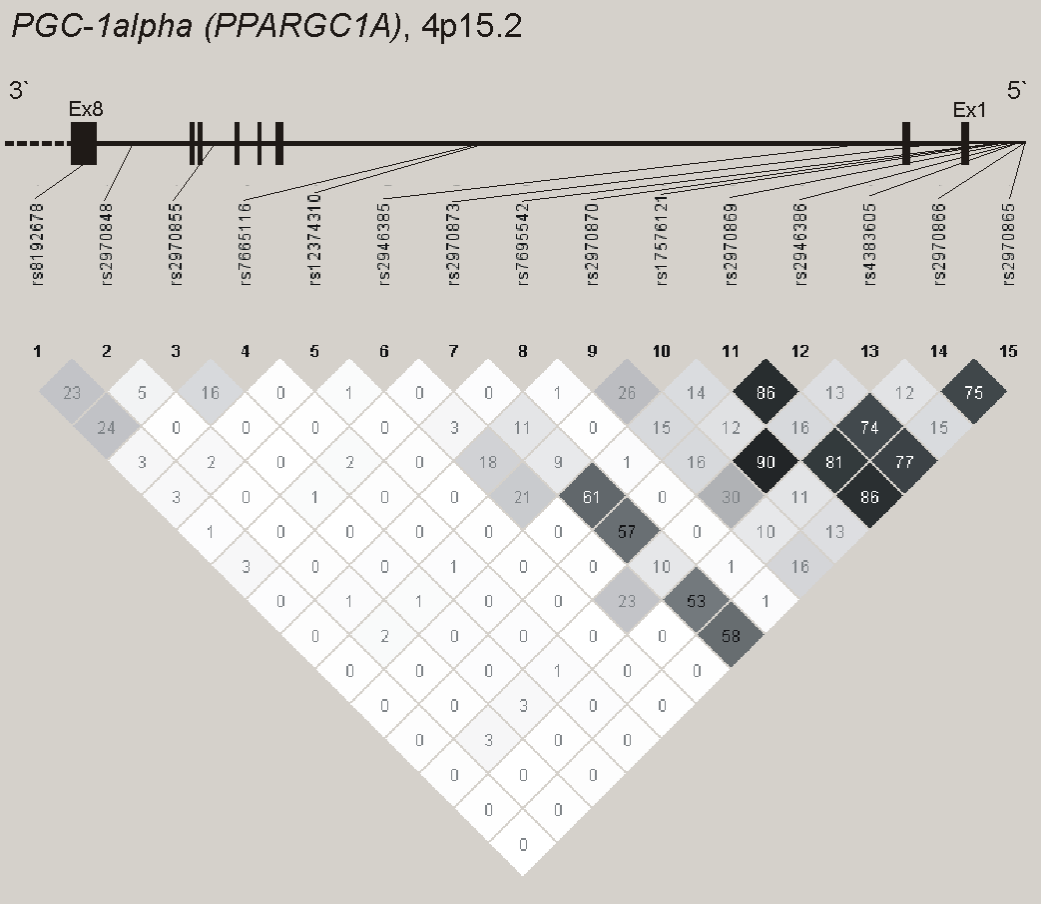
**Schematic representation of the *PGC-1alpha *gene and linkage disequilibrium (r2) in the analysed HD cohort**. Black boxes and a horizontal line represent exons and introns respectively. The numbers in the cells denote the r^2 ^between the two SNPs corresponding to the cell. Cell shading indicates strength of r^2 ^as shown by the number.

Figure 1 shows the HapMap r^2 ^values among these SNPs in the HD cohort and reflects the previously reported rough subdivision into 2 main haplotype blocks [20]. The first one includes the polymorphisms in the promoter region and intron 1 (rs2970865, rs2970866, rs4383605, rs2946386, rs2970869, rs17576121, rs2970870, rs7695542, rs2970873), whereas the second includes the SNPs located 3' downstream of rs7665116 in intron 2. The two haplotype blocks are separated by a region of high recombination frequency. These results comply with the HapMap database.

The rs7665116 T > C polymorphism is located within intron 2, a potential recombination hot spot. Sequence alignments of multiple species show that this SNP is located at the beginning of a 233 bp highly conserved sequence. Yet, the rs7665116 polymorphism itself is not conserved to any significant extent across species. No other SNPs are described in this conserved sequence.

*In silico*-analysis of rs7665116 using MatInspector [21] revealed loss of potential binding sites for cAMP-responsive element binding proteins (V$CHOP.01) in case of the C allele as compared to the wildtype T allele. On the other hand, in case of the C allele a new binding site is generated for v-Myb (V$VMYB.02) and X-box binding protein RFX1 (V$RFX1.01). Yet, it can only be speculated that the conserved region around rs7665116 represents a regulatory region controlling constitutive functions of *PGC-1alpha*. Functional studies are needed to assess whether *PGC-1alpha *is a true modifier gene and to identify the causal genetic variations contributing in the pathogenesis of HD in this region.

While this manuscript was under review, an article by Weydt *et al. *was published in this journal showing a modifying effect of *PGC-1alpha *haploblock 2 variations upon AO in an Italian cohort of 447 unrelated HD patients [22]. Our independent confirmation of their findings in a German cohort strengthens the conclusion that the *PGC-1alpha *gene appears to have modifying effects on the pathogenic process in HD and that it may be a therapeutically useful target for development of a treatment. Yet, it will be necessary to delineate of the precise basis for the *PGC-1alpha *modifier effect in order to effectively undertake a search for chemical compounds that delay HD onset.

## Abbreviations

PGC-1*alpha*: peroxisome proliferator-activated receptor gamma co-activator

## Competing interests

The authors declare that they have no competing interests.

## Authors' contributions

ETF carried out the molecular genetic studies and helped to design the study and draft the manuscript. CS and JA had ascertained the clinical status of the patients. SW interpreted the data and reviewed the manuscript. LA designed the study including statistical analysis and drafted the manuscript.
